# Cinnamoyl­thio­urea

**DOI:** 10.1107/S1600536810040018

**Published:** 2010-10-13

**Authors:** Ibrahim N. Hassan, Bohari M. Yamin, Mohammad B. Kassim

**Affiliations:** a School of Chemical Sciences and Food Technology, Faculty of Science and Technology, Universiti Kebangsaan Malaysia, UKM 43600 Bangi Selangor, Malaysia

## Abstract

In the title compound [systematic name: 1-(3-phenyl­prop-2-eno­yl)thio­urea], C_10_H_10_N_2_OS, the acetyl­thio­urea fragment and the phenyl ring adopt an *E* configuration. The roughly planar but-2-enoyl­thio­urea fragment [maximum deviation = 0.053 (3) Å] forms a dihedral of 10.54 (11)° with the phenyl ring. An intra­molecular N—H⋯O hydrogen bond generates an *S*(6) ring. In the crystal, mol­ecules are linked into sheets parallel to (100) by N—H⋯S hydrogen bonds.

## Related literature

For the preparation, see: Hassan *et al.* (2010*a* ▶). For related structures, see: Hung *et al.* (2010 ▶); Hassan *et al.* (2008**a* ▶,*b* ▶,c* ▶, 2009 ▶, 2010**a* ▶,b* ▶); Yamin & Hassan (2004 ▶). For bond-length data, see: Allen *et al.* (1987 ▶).
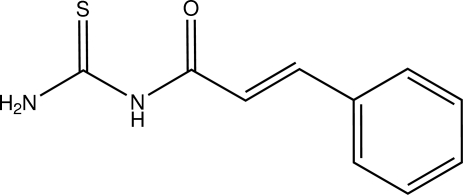


## Experimental

### 

#### Crystal data

C_10_H_10_N_2_OS
*M* *_r_* = 206.26
Monoclinic, 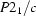

*a* = 14.3313 (18) Å
*b* = 4.9801 (6) Å
*c* = 15.4199 (19) Å
β = 111.612 (3)°
*V* = 1023.2 (2) Å^3^
*Z* = 4
Mo *K*α radiation
μ = 0.28 mm^−1^
*T* = 273 K
0.34 × 0.12 × 0.09 mm

#### Data collection

Bruker SMART APEX CCD area-detector diffractometer
Absorption correction: multi-scan (*SADABS*; Sheldrick, 2000 ▶) *T*_min_ = 0.960, *T*_max_ = 0.975
7192 measured reflections
2551 independent reflections
1688 reflections with *I* > 2σ(*I*)
*R*_int_ = 0.039

#### Refinement

*R*[*F* ^2^ > 2σ(*F* ^2^)] = 0.065
*wR*(*F* ^2^) = 0.156
*S* = 1.08
2551 reflections
127 parameters
3 restraints
H-atom parameters constrained
Δρ_max_ = 0.40 e Å^−3^
Δρ_min_ = −0.20 e Å^−3^

### 

Data collection: *SMART* (Bruker, 2000 ▶); cell refinement: *SAINT* (Bruker, 2000 ▶); data reduction: *SAINT*; program(s) used to solve structure: *SHELXS97* (Sheldrick, 2008 ▶); program(s) used to refine structure: *SHELXL97* (Sheldrick, 2008 ▶); molecular graphics: *SHELXTL* (Sheldrick, 2008 ▶); software used to prepare material for publication: *SHELXTL*, *PARST* (Nardelli, 1995 ▶) and *PLATON* (Spek, 2009 ▶).

## Supplementary Material

Crystal structure: contains datablocks global, I. DOI: 10.1107/S1600536810040018/ci5180sup1.cif

Structure factors: contains datablocks I. DOI: 10.1107/S1600536810040018/ci5180Isup2.hkl

Additional supplementary materials:  crystallographic information; 3D view; checkCIF report

## Figures and Tables

**Table 1:** Hydrogen-bond geometry (Å, °)

*D*—H⋯*A*	*D*—H	H⋯*A*	*D*⋯*A*	*D*—H⋯*A*
N2—H2*A*⋯O1	0.89	1.95	2.649 (3)	134
N1—H1*A*⋯S1^i^	0.85	2.79	3.602 (2)	159
N2—H2*B*⋯S1^ii^	0.88	2.54	3.409 (2)	169

Symmetry codes: (i) 
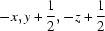
; (ii) 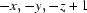.

**Fig. 1.**
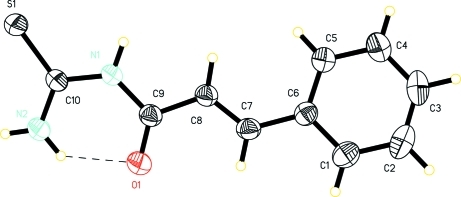
The molecular structure of (I), with displacement ellipsoids drawn at the 50% probability level.

**Fig. 2.**
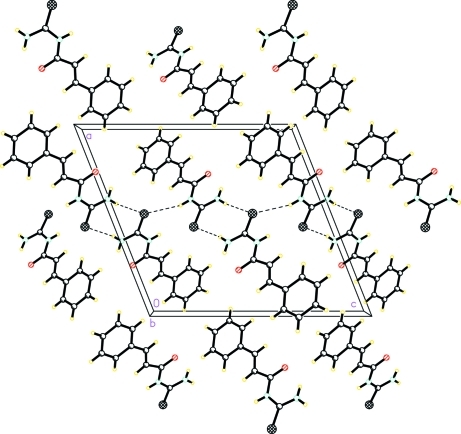
Part of the crystal packing of (I), viewed down the b axis. Hydrogen bonds are shown as dashed lines.

## supplementary crystallographic information

### Comment

The title compound, (I), is an amide thiourea derivative of cinnamoyl analogous to our previously reported molecules, methyl-2-(3-cinnamoylthioureido)acetate (Hassan *et al.*, 2010*a*) (II), propyl-2-(3-benzoylthioureido)acetate (Hassan *et al.*, 2008*b*) (III), butyl-2-(3-benzoylthioureido)acetate (Hassan *et al.*, 2008*c*) (IV), methyl-2-(3-benzoylthioureido)acetate (Hassan *et al.*, 2009) (V) and ethyl-2-(3-cinnamoylthioureido)acetate (Hassan *et al.*, 2010*b*) (VI). As in most of carbonylthiourea derivatives of the type *R*^1^C(O)NHC(S)NH*R*^2^, the molecule maintains the *E—Z* configuration with respect to the positions of the cinnamoyl moiety and the hydrogen atom of the terminal amide group, respectively, relative to the S atom across the C10—N2 bond (Fig 1). The bond lengths (Allen *et al.*, 1987) and angles in the molecule are in normal ranges and comparable to those observed in (II), (III), (IV), (V) and (VI). However, the C═S bond length [1.679 (2) Å] is slightly longer than that observed in (II) [1.666 (3) Å]. The S1/O1/N1/N2/C6/C7/C8/C9/C10 fragment is essentially planar with a maximum deviation of 0.053 (3) Å, for atom C8. The C1–C6 phenyl ring is inclined to the above plane with a dihedral angle of 10.54 (11)°, which is smaller than that found in (II) [11.17 (14)°]. There is one intramolecular hydrogen bond, N2—H2A^···^O1 (Table 1), which resulted in the formation of pseudo-six-membered ring (N2/H2A/O1/C9/N1/C10) (Fig 1). The molecular packing is stablized by two intermolecular hydrogen bonds viz. N1—H1A^···^S1 and N1—H1A^···^S1 (Table 1) which form a two-dimensional network parallel to (100) [Fig. 2].

### Experimental

The title compound was obtained from a reaction sheme similar to the cinnamoylthiourea ester synthesis starting from 2-(3-cinnamoylthioureido) acetic acid with alcohol in the presence of LaCl_3_ reported earlier (Hassan *et al.*, 2010*a*). Colourless single crystals, suitable for X-ray analysis, were obtained by slow evaporation of a CH_2_Cl_2_ solution at room temperature (yield 77%).

### Refinement

H atoms of C atoms were positioned geometrically [C–H = 0.93 Å] and allowed to ride on their parent atoms, with *U*_iso_ = 1.2*U*_eq_(C). The H atoms of N atoms were located in difference fouriuor maps and allowed to ride on their parent atoms, with N–H = 0.87 (1) Å and *U*_iso_ = 1.2*U*_eq_(N).

### Crystal data

**Table:**

C_10_H_10_N_2_OS	*F*(000) = 432
*M**_r_* = 206.26	*D*_x_ = 1.339 Mg m^−^^3^
Monoclinic, *P*2_1_/*c*	Mo *K*α radiation, λ = 0.71073 Å
Hall symbol: -P 2ybc	Cell parameters from 1059 reflections
*a* = 14.3313 (18) Å	θ = 1.5–28.3°
*b* = 4.9801 (6) Å	µ = 0.28 mm^−^^1^
*c* = 15.4199 (19) Å	*T* = 273 K
β = 111.612 (3)°	Plate, colourless
*V* = 1023.2 (2) Å^3^	0.34 × 0.12 × 0.09 mm
*Z* = 4	

### Data collection

**Table:**

Bruker SMART APEX CCD area-detector diffractometer	2551 independent reflections
Radiation source: fine-focus sealed tube	1688 reflections with *I* > 2σ(*I*)
graphite	*R*_int_ = 0.039
ω scans	θ_max_ = 28.3°, θ_min_ = 1.5°
Absorption correction: multi-scan (*SADABS*; Sheldrick, 2000)	*h* = −18→19
*T*_min_ = 0.960, *T*_max_ = 0.975	*k* = −6→6
7192 measured reflections	*l* = −20→16

### Refinement

**Table:**

Refinement on *F*^2^	Primary atom site location: structure-invariant direct methods
Least-squares matrix: full	Secondary atom site location: difference Fourier map
*R*[*F*^2^ > 2σ(*F*^2^)] = 0.065	Hydrogen site location: inferred from neighbouring sites
*wR*(*F*^2^) = 0.156	H-atom parameters constrained
*S* = 1.08	*w* = 1/[σ^2^(*F*_o_^2^) + (0.0703*P*)^2^ + 0.1088*P*] where *P* = (*F*_o_^2^ + 2*F*_c_^2^)/3
2551 reflections	(Δ/σ)_max_ = 0.001
127 parameters	Δρ_max_ = 0.40 e Å^−^^3^
3 restraints	Δρ_min_ = −0.20 e Å^−^^3^

### Special details

**Table:**

Geometry. All e.s.d.'s (except the e.s.d. in the dihedral angle between two l.s. planes) are estimated using the full covariance matrix. The cell e.s.d.'s are taken into account individually in the estimation of e.s.d.'s in distances, angles and torsion angles; correlations between e.s.d.'s in cell parameters are only used when they are defined by crystal symmetry. An approximate (isotropic) treatment of cell e.s.d.'s is used for estimating e.s.d.'s involving l.s. planes.
Refinement. Refinement of *F*^2^ against ALL reflections. The weighted *R*-factor *wR* and goodness of fit *S* are based on *F*^2^, conventional *R*-factors *R* are based on *F*, with *F* set to zero for negative *F*^2^. The threshold expression of *F*^2^ > σ(*F*^2^) is used only for calculating *R*-factors(gt) *etc*. and is not relevant to the choice of reflections for refinement. *R*-factors based on *F*^2^ are statistically about twice as large as those based on *F*, and *R*- factors based on ALL data will be even larger.

### Fractional atomic coordinates and isotropic or equivalent isotropic displacement parameters (Å^2^)

**Table:**

	*x*	*y*	*z*	*U*_iso_*/*U*_eq_	
S1	−0.03467 (5)	0.16643 (15)	0.35571 (4)	0.0559 (3)	
O1	0.24962 (15)	0.6309 (4)	0.51663 (12)	0.0647 (6)	
N1	0.11111 (14)	0.5226 (4)	0.38961 (13)	0.0428 (5)	
H1A	0.0790	0.5740	0.3339	0.051*	
N2	0.11216 (16)	0.2739 (5)	0.51525 (14)	0.0551 (6)	
H2A	0.1680	0.3641	0.5479	0.066*	
H2B	0.0850	0.1571	0.5415	0.066*	
C1	0.2743 (2)	1.2916 (6)	0.26352 (19)	0.0553 (7)	
H1B	0.2112	1.2149	0.2350	0.066*	
C2	0.3067 (2)	1.4824 (6)	0.2166 (2)	0.0674 (8)	
H2C	0.2655	1.5331	0.1567	0.081*	
C3	0.3993 (2)	1.5982 (6)	0.2575 (2)	0.0676 (8)	
H3A	0.4206	1.7273	0.2253	0.081*	
C4	0.4604 (2)	1.5251 (6)	0.3454 (2)	0.0669 (8)	
H4A	0.5232	1.6043	0.3731	0.080*	
C5	0.42846 (19)	1.3317 (6)	0.3932 (2)	0.0551 (7)	
H5A	0.4704	1.2819	0.4530	0.066*	
C6	0.33484 (18)	1.2118 (5)	0.35306 (17)	0.0431 (6)	
C7	0.30347 (18)	1.0120 (5)	0.40557 (17)	0.0456 (6)	
H7A	0.3462	0.9838	0.4670	0.055*	
C8	0.22069 (18)	0.8664 (5)	0.37521 (16)	0.0434 (6)	
H8A	0.1758	0.8900	0.3143	0.052*	
C9	0.19735 (18)	0.6684 (5)	0.43484 (16)	0.0433 (6)	
C10	0.06860 (17)	0.3267 (5)	0.42595 (15)	0.0395 (5)	

### Atomic displacement parameters (Å^2^)

**Table:**

	*U*^11^	*U*^22^	*U*^33^	*U*^12^	*U*^13^	*U*^23^
S1	0.0556 (4)	0.0654 (5)	0.0402 (4)	−0.0166 (3)	0.0101 (3)	0.0071 (3)
O1	0.0744 (13)	0.0688 (13)	0.0390 (10)	−0.0198 (10)	0.0072 (9)	0.0076 (9)
N1	0.0475 (11)	0.0421 (11)	0.0342 (10)	−0.0031 (9)	0.0097 (9)	0.0060 (9)
N2	0.0651 (14)	0.0608 (15)	0.0354 (11)	−0.0148 (11)	0.0139 (10)	0.0072 (10)
C1	0.0558 (15)	0.0559 (17)	0.0509 (15)	−0.0097 (13)	0.0160 (13)	0.0026 (13)
C2	0.077 (2)	0.065 (2)	0.0623 (18)	−0.0090 (17)	0.0280 (16)	0.0113 (16)
C3	0.081 (2)	0.0569 (18)	0.083 (2)	−0.0114 (16)	0.0516 (19)	0.0027 (17)
C4	0.0598 (17)	0.0601 (19)	0.092 (2)	−0.0174 (15)	0.0407 (18)	−0.0169 (18)
C5	0.0523 (15)	0.0526 (16)	0.0576 (17)	−0.0040 (13)	0.0170 (13)	−0.0088 (14)
C6	0.0444 (12)	0.0373 (13)	0.0470 (14)	−0.0009 (10)	0.0162 (11)	−0.0036 (11)
C7	0.0488 (14)	0.0427 (14)	0.0392 (12)	−0.0002 (11)	0.0090 (11)	−0.0021 (11)
C8	0.0490 (13)	0.0410 (14)	0.0368 (12)	−0.0022 (11)	0.0117 (10)	−0.0003 (11)
C9	0.0504 (13)	0.0408 (14)	0.0369 (13)	−0.0005 (11)	0.0140 (11)	−0.0009 (11)
C10	0.0455 (12)	0.0383 (12)	0.0360 (12)	0.0041 (11)	0.0162 (10)	0.0034 (10)

### Geometric parameters (Å, °)

**Table:**

S1—C10	1.679 (2)	C2—H2C	0.93
O1—C9	1.221 (3)	C3—C4	1.365 (4)
N1—C10	1.374 (3)	C3—H3A	0.93
N1—C9	1.381 (3)	C4—C5	1.389 (4)
N1—H1A	0.85	C4—H4A	0.93
N2—C10	1.312 (3)	C5—C6	1.389 (3)
N2—H2A	0.89	C5—H5A	0.93
N2—H2B	0.88	C6—C7	1.455 (3)
C1—C2	1.374 (4)	C7—C8	1.320 (3)
C1—C6	1.391 (3)	C7—H7A	0.93
C1—H1B	0.93	C8—C9	1.468 (3)
C2—C3	1.369 (4)	C8—H8A	0.93
			
C10—N1—C9	128.04 (19)	C6—C5—C4	121.0 (3)
C10—N1—H1A	118.0	C6—C5—H5A	119.5
C9—N1—H1A	113.7	C4—C5—H5A	119.5
C10—N2—H2A	118.2	C5—C6—C1	117.8 (2)
C10—N2—H2B	119.8	C5—C6—C7	119.5 (2)
H2A—N2—H2B	122.0	C1—C6—C7	122.7 (2)
C2—C1—C6	120.8 (3)	C8—C7—C6	126.9 (2)
C2—C1—H1B	119.6	C8—C7—H7A	116.6
C6—C1—H1B	119.6	C6—C7—H7A	116.6
C3—C2—C1	120.4 (3)	C7—C8—C9	121.9 (2)
C3—C2—H2C	119.8	C7—C8—H8A	119.0
C1—C2—H2C	119.8	C9—C8—H8A	119.0
C4—C3—C2	120.3 (3)	O1—C9—N1	122.4 (2)
C4—C3—H3A	119.9	O1—C9—C8	123.7 (2)
C2—C3—H3A	119.9	N1—C9—C8	113.9 (2)
C3—C4—C5	119.7 (3)	N2—C10—N1	117.3 (2)
C3—C4—H4A	120.1	N2—C10—S1	123.18 (19)
C5—C4—H4A	120.1	N1—C10—S1	119.49 (17)

### Hydrogen-bond geometry (Å, °)

**Table:**

*D*—H···*A*	*D*—H	H···*A*	*D*···*A*	*D*—H···*A*
N2—H2A···O1	0.89	1.95	2.649 (3)	134
N1—H1A···S1^i^	0.85	2.79	3.602 (2)	159
N2—H2B···S1^ii^	0.88	2.54	3.409 (2)	169

Symmetry codes:  (i) −*x*, *y*+1/2, −*z*+1/2; (ii) −*x*, −*y*, −*z*+1.
